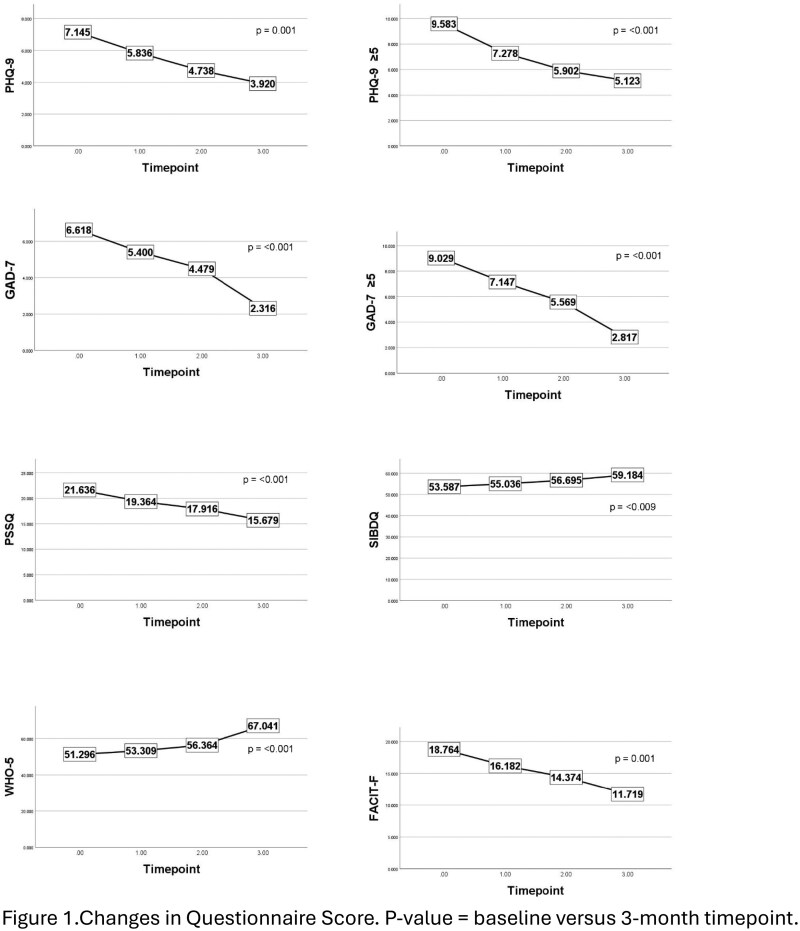# Poster Session I - A153 PRELIMINARY RESULTS ON THE EFFECTIVENESS OF A MENTAL HEALTH PATHWAY FOR IBD

**DOI:** 10.1093/jcag/gwaf042.153

**Published:** 2026-02-13

**Authors:** E Rahime, B Elchitz, K Wong, R Kaur, S Raiesdana, K D Chappell, Y Zhang, F Peerani, K Kroeker, D Kao, M Gozdzik, F Hoentjen, B Halloran, C Seow

**Affiliations:** University of Alberta Department of Medicine, Edmonton, AB, Canada; University of Calgary, Calgary, AB, Canada; University of Alberta Department of Medicine, Edmonton, AB, Canada; University of Alberta Department of Medicine, Edmonton, AB, Canada; University of Alberta Department of Medicine, Edmonton, AB, Canada; University of Alberta Department of Medicine, Edmonton, AB, Canada; University of Alberta Department of Medicine, Edmonton, AB, Canada; University of Alberta Department of Medicine, Edmonton, AB, Canada; University of Alberta Department of Medicine, Edmonton, AB, Canada; University of Alberta Department of Medicine, Edmonton, AB, Canada; University of Alberta Department of Medicine, Edmonton, AB, Canada; University of Alberta Department of Medicine, Edmonton, AB, Canada; University of Alberta Department of Medicine, Edmonton, AB, Canada; University of Calgary, Calgary, AB, Canada

## Abstract

**Background:**

Patients with inflammatory bowel disease (IBD) experience high rates of depression, anxiety, and stress, negatively affecting quality of life, disease activity, and health care utilization. Despite this, mental health care remains under-integrated in IBD care.

**Aims:**

The aim of this study is to evaluate the effectiveness of a mental health pathway for IBD, consisting of screening for anxiety and depression and incorporating digital tools for self-care.

**Methods:**

This prospective, single-arm intervention study was conducted at two academic centers. Those who entered the pathway were introduced to digital self-management tools and completed questionnaires at baseline, 1, 2, and 3 months. Outcomes included depression (PHQ-9), anxiety (GAD-7), stress (PSSQ), fatigue (FACIT-F), quality of life (SIBDQ, WHO-5), and work productivity (WPAI). Linear mixed models evaluated longitudinal changes in participants who completed the 1-month follow-up, and Mann–Whitney U tests compared outcomes by disease activity.

**Results:**

116 participants were enrolled between May and Sept 2025. At 1 month, 56 participants (62.5% female, range 18-74) completed follow-up, 35 at 2 months, and 15 at 3 months. PHQ-9 improved across timepoints , with a 3.2-point reduction by 3 months. GAD-7 improved at all timepoints, with reductions of 4.3 points by 3 months. Participants with baseline PHQ-9 or GAD-7 ≥ 5 in comparison to < 5 had greater improvement at each time point. On average, PHQ-9 scores decreased by 45.1% overall and 46.5% among those with baseline ≥5, while GAD-7 scores decreased by 65.0% overall and 68.8% among those with baseline ≥5, exceeding established minimal clinically important difference (MCID) thresholds (≥5-point or ≥ 20% reduction). PSSQ decreased at all timepoints, FACIT-F improved at all timepoints, SIBDQ improved at 2 and 3 months, and WHO-5 at 3 months. No effects were seen for WPAI.

At 1 month, patients with self-reported active disease showed higher anxiety (p = .016), stress (p = .005), fatigue (p = .050), and lower SIBDQ (p = .009) and WHO-5 (p = .008). At 2 months, active disease was linked to higher depression (p = .021), lower SIBDQ (p = .004), and higher fatigue (p = .044).

**Conclusions:**

Interim results indicate that digital self-care tools were associated with clinically meaningful short-term improvements in depression, anxiety, stress, fatigue, and quality of life. Average reductions in PHQ-9 and GAD-7 scores exceeded published MCID thresholds, suggesting that observed changes reflect meaningful symptom improvement. Long-term outcomes (>3 months) remain to be evaluated.

**Funding Agencies:**

Alberta Innovates, Northern Alberta Clinical Trials and Research Centre